# Morphostructural Analysis of PAH-Rich Human Adipose Tissue: A Potential Silent Sequestration Site

**DOI:** 10.3390/ijms27125607

**Published:** 2026-06-21

**Authors:** Elena Stocco, Silvia Barbon, Martina Contran, Valentina Manzo, Daniele Brunelli, Luca Sorarù, Alice Franchin, Elena Gregoris, Marco Roman, Andrea Gambaro, Warren R. L. Cairns, Raffaele De Caro, Vincenzo Vindigni, Veronica Macchi, Andrea Porzionato

**Affiliations:** 1 Department of Neuroscience, Section of Human Anatomy, University of Padova, via Aristide Gabelli 65, 35121 Padua, Italy; silvia.barbon@unipd.it (S.B.); martina.contran@unipd.it (M.C.); valentina.manzo@unipd.it (V.M.); raffaele.decaro@unipd.it (R.D.C.); andrea.porzionato@unipd.it (A.P.); 2 Department of Women’s and Children’s Health, University of Padova, via Nicolò Giustiniani 3, 35128 Padua, Italy; 3 Department of Surgery, Oncology and Gastroenterology, University of Padova, via Nicolò Giustiniani 2, 35124 Padua, Italy; 4 Reference Center for the Preservation and Use of Bodies, Veneto Region, via Aristide Gabelli 65, 35121 Padua, Italy; 5 Clinic of Plastic, Reconstructive and Aesthetic Surgery, Department of Neuroscience, University Hospital of Padua, via Nicolò Giustiniani 2, 35128 Padua, Italy; daniele.brunelli@studenti.unipd.it (D.B.); vincenzo.vindigni@unipd.it (V.V.); 6 Department of Environmental Sciences, Informatics and Statistics, Ca’ Foscari University of Venice, via Torino 155, 30172 Venice Mestre, Italy; luca.soraru@unive.it (L.S.); alice.franchin@unive.it (A.F.); marco.roman@unive.it (M.R.); gambaro@unive.it (A.G.); 7 Institute of Polar Sciences, National Research Council (ISP-CNR), via Torino 155, 30172 Venice, Italy; elena.gregoris@cnr.it (E.G.); warrenraymondlee.cairns@cnr.it (W.R.L.C.)

**Keywords:** polycyclic aromatic hydrocarbons, pyrene, toxic agents, adipose tissue, adipocytes, reservoir, morpho-structure, connective tissue, inflammation

## Abstract

Polycyclic aromatic hydrocarbons (PAHs) are widespread, persistent pollutants that can be sequestered within human adipose tissue due to their lipophilic nature. While this accumulation poses toxicological risks depending on dose and individual susceptibility, the specific morphological impact of chronic PAH storage on tissue architecture remains poorly defined. Here, we performed a histopathological and morphometric analysis on human subcutaneous adipose tissue samples characterized by high pyrene levels. We evaluated tissue organization, collagen distribution, the presence of inflammatory, neural, and vascular alterations and adipocyte morphometry to assess the structural response to PAH sequestration. Despite high pyrene concentrations, PAH-positive tissues maintained preserved overall architecture with normal collagen distribution, absence of lymphocytic infiltration, low macrophages, unaltered nerve fiber patterns, without evidence of vascular remodeling. Morphometry revealed smaller adipocyte area in PAH-positive samples, although not statistically significant. Our experimental data indicate that high PAH accumulation does not necessarily induce subcutaneous adipose tissue remodeling, suggesting that biochemical or metabolic alterations might occur even in the absence of evident histological changes. Further studies, with a broadened cohort, are needed to define the threshold at which PAHs’ presence translates into permanent tissue damage.

## 1. Introduction

Polycyclic aromatic hydrocarbons (PAHs) are a class of widespread persistent organic pollutants that mainly derive from the incomplete combustion of organic matter from both natural sources (e.g., volcanic eruptions and forest fires) and anthropogenic activities (e.g., industrial emissions, vehicle exhaust, residential heating, and tobacco smoke) [1,2]. Moreover, PAHs can be detected in different types/concentrations during food processing [3]. Due to their high toxicity, PAHs represent a significant threat for both ecosystems and human health, entering the human body through multiple ways that include inhalation, ingestion, or dermal absorption [4]. As air intake per day ranges from 3.6 to 16.3 m^3^ (according to subject age), inhalation is certainly a continuous and stable source of exposure [1]. Ingestion is influenced by diet; thus, it is more variable. Food contamination can derive from environmental deposition or high-temperature cooking, processing, and preservation methods (drying and curing) [3]. Finally, dermal absorption may also occur, but it is minimal, because of the limited area of contact and slow penetration rate through the skin [1]. As environmental pollution is a global burden that continues to rise, the potential metabolic and cellular effects of PAHs on human health are gaining increasing attention. Different adverse effects can be attributed to PAHs exposure including carcinogenic and mutagenic effects; moreover, they are potent immune suppressants [2,5]. Certainly, the health risks associated with PAHs depend on multiple factors, including the toxicity of specific compounds, individual susceptibility to a specific contaminant, and most importantly, the duration of exposure; this latter is considered the key determinant of adverse outcomes [2].

In the human body, white adipose tissue (WAT) is a metabolically active endocrine organ representing the primary energy reservoir, in the form of lipids. It also plays a fundamental role in maintaining whole-body energy homeostasis through the secretion of hormones, adipokines, and pro-inflammatory mediators which influence both metabolic and immune functions [6,7]. Due to its high lipid content, adipose tissue can act as a long-term reservoir for lipophilic environmental toxic agents [8,9], which are slowly released into circulation during triglyceride lipolysis [10]. Among these lipophilic pollutants are PAHs [11]. However, the specific effects of chronic PAH accumulation on adipose tissue remain poorly understood, and studies investigating potential morpho-structural alterations are still limited, highlighting an important knowledge gap [9].

To contribute to this field, in this work we analyzed the morpho-structural characteristics of human-derived subcutaneous adipose tissue that had been previously assessed for PAH presence (pyrene detected) at concentrations higher (from 2.34 to 4.88 ng/g) [12] than those reported in the literature (lower than 0.252 ng/g) [9,10,13]. The samples were examined by light microscopy (haematoxylin and eosin staining, H&E; Masson’s Trichrome staining), immunohistochemical analyses (CD3, lymphocytes; CD68, macrophages; S100 and PGP9.5, nerve endings; CD31 and VCAM-1, endothelial cells), and morphometric evaluation (adipocyte area) to assess potential histopathological modifications attributable to PAH exposure. Given the broad human exposure to PAHs, elucidating its impact on adipose tissue at the morphological and cellular levels could provide valuable insights into the environmental influences on tissue remodeling and dysfunction.

## 2. Results

### 2.1. Adipose Tissue Organization and Extracellular Matrix

**Figure 1:**
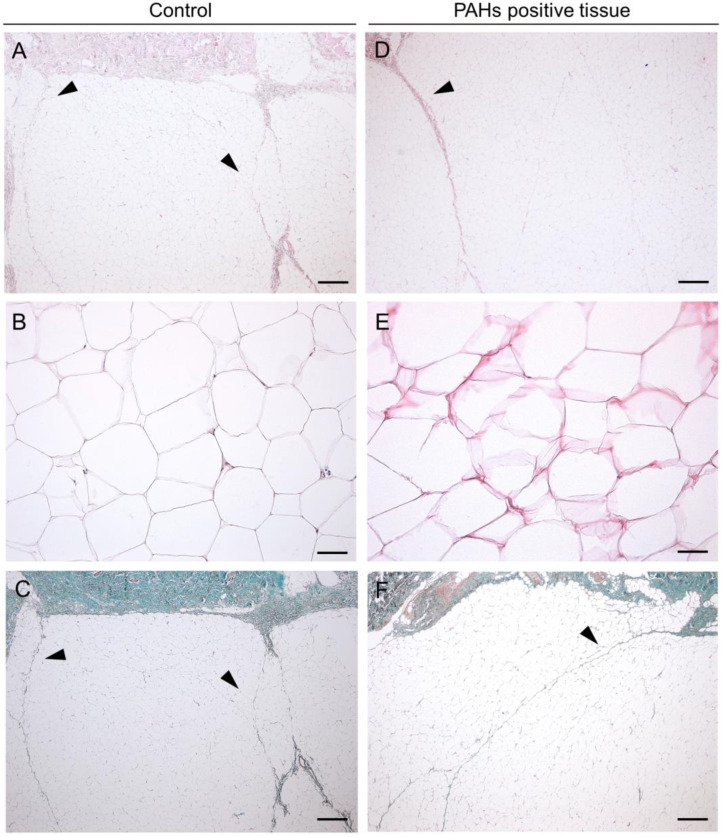
Hematoxylin and eosin–stained sections (**A**,**B**,**D**,**E**) and Masson’s Trichrome (**C**,**F**) stained sections showing tissue architecture and adipocyte morphology in control and PAH-positive specimens. Thin septa (black arrows) originate from the deep dermis/hypodermis interface and extend into the hypodermis, delineating adipocyte compartments. Scale bars: 400 µm (**A**,**C**,**D**,**F**); 50 µm (**B**,**E**).

Subcutaneous adipose tissue architecture was assessed in all specimens using H&E staining and Masson’s Trichrome staining for collagen visualization (Figure 1A–F). At the deep dermis–hypodermis interface, connective tissue was characterized by increased collagen deposition. Thin fibrous strands extended from the deep dermis into the hypodermis, forming fine connective tissue septa, delineating adipocyte lobules. Adipocytes exhibited the expected round to polygonal morphology, with large cytoplasm and small, flattened nuclei positioned at the cell periphery. No appreciable differences in overall tissue organization or adipocyte morphology were observed between PAH-positive samples and the PAH-negative specimen.

### 2.2. Immunodetection of Inflammation-Related Elements

**Figure 2:**
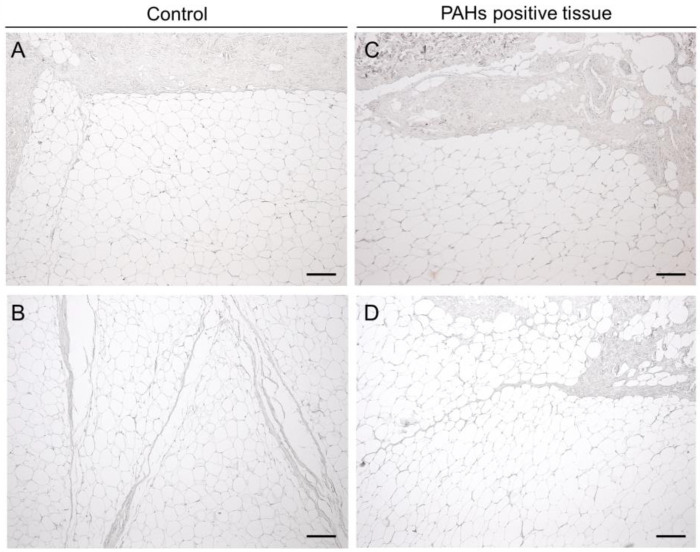
Representative images showing CD3 immunodetection in a control sample and in specimens positive for PAHs. Focus is given to the deep dermis/hypodermis interface (**A**,**C**) and the overall appearance of the hypodermis (**B**,**D**). No positive elements were observed. No CD3-positive elements were detected in any of the samples. Scale bar: 200 µm.

Immunodetection of CD3-positive elements was negative across the entire cohort. Based on the scoring criteria, all specimens were assigned a grade of 0, indicating the absence of T-lymphocytic infiltration at the deep dermis/hypodermis interface and within the hypodermis (Figure 2). No local accumulations or scattered CD3-positive cells were detected.

**Figure 3:**
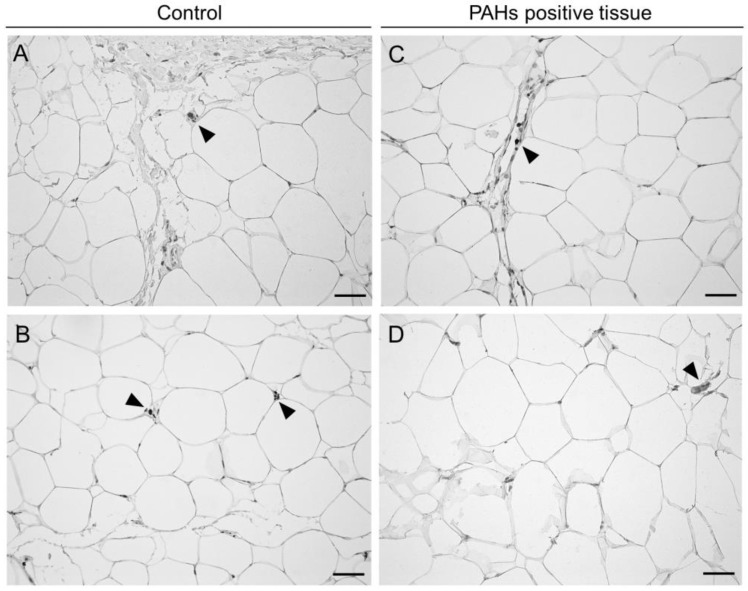
Representative images showing CD68 immunodetection in a control sample and in specimens positive for PAHs. Immuno-positive elements, recognizable within septa, in correspondence of vessels or among adipocytes, are highlighted by the black arrows. Scale bars: 100 µm (**A**,**C**); 50 µm (**B**,**D**).

The presence of macrophages was assessed through CD68 immunostaining. As shown in Figure 3, few positive elements were detected across the entire cohort, localized within connective septa, adjacent to blood vessels, or as isolated cells among adipocytes. The distribution of CD68-positive elements did not differ between PAH-positive tissues and the control specimen.

### 2.3. Nerve Endings and Vessels Detection

**Figure 4:**
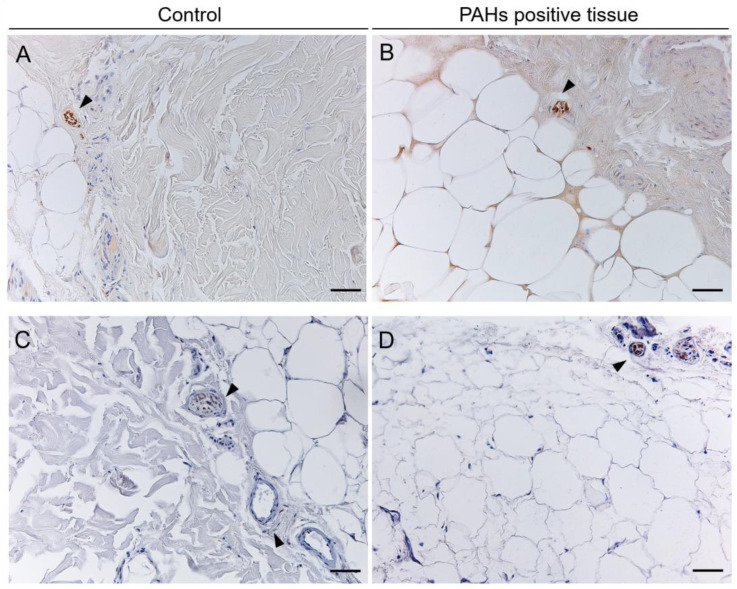
Representative images showing S100 (**A**,**B**) and PGP9.5 (**C**,**D**) immunodetection in a control sample and in specimens positive for PAHs. Black arrows show nerves and nerve endings. Scale bars: 50 µm.

S100 and PGP9.5 immunohistochemical analysis revealed the presence of nerve endings in the deep dermis of the whole cohort. Based on the samples investigated, positive elements appeared sparse and rare, typically at the interface with the hypodermis. No qualitative differences in the density or localization of immuno-positive nerve endings were observed between PAH-positive specimens and the PAH-negative control (Figure 4).

**Figure 5:**
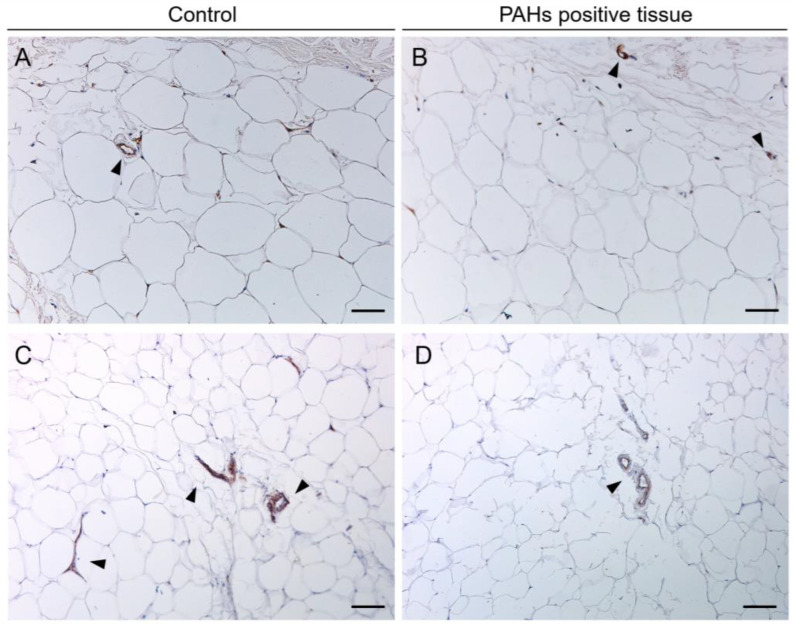
Representative images showing CD31 (**A**,**B**) and VCAM-1 (**C**,**D**) immunodetection in a control sample and in specimens positive for PAHs. Immuno-positive elements are highlighted by the black arrows. Scale bars: 50 µm (**A**,**B**); 100 µm (**C**,**D**).

Vessels and CD31-immunopositive cells were similarly distributed in both control and PAH-positive tissues (Figure 5A,B). In both the deep dermis, particularly at the interface with the hypodermis, and within the hypodermis itself, these elements appeared sparse and often associated with fibrous strands, although not exclusively. No differences in vessel density or arrangement emerged between PAH-positive specimens and the control.

VCAM-1-positive elements were also assessed. VCAM-1 immunoreactivity was localized to the vascular compartment in both control and PAH samples, with no differences in staining distribution between the two groups (Figure 5C,D).

### 2.4. Adipocytes Morphometric Study

The morphometric analysis of adipocyte area allowed for comparison among specimens and between PAH-positive samples versus the PAH-negative sample. The calculated values (mean values ± SD, µm^2^) and significances are reported in Table 1. Considering the control specimen, significantly lower values were detected compared to samples #9 (*p* ≤ 0.01), #5 (*p* ≤ 0.001) and #6 (*p* ≤ 0.0001).

Overall, lower mean values were measured for PAH-positive samples (6901.31 ± 1519.84 µm^2^) compared with the control sample (7633.70 ± 2053.21 µm^2^). However, this difference did not reach statistical significance.

## 3. Discussion

Pyrene is a PAH commonly present in environmental pollutants. It is not classified as a confirmed human carcinogen, but it is considered a potential chemical carcinogen and tumor-initiating compound because of a structure that is similar to that of other carcinogenic PAHs [14]. As the adipose tissue behaves as a reservoir for lipophilic substances [15] (including carcinogens and their initiators), it has an important toxicological role by sequestering these compounds and protecting other more sensitive lipophilic organs (like the brain) from an overload [16]. Specifically, pyrene was selected as the representative target for quantification due to its stability and ubiquity in various environmental combustion sources. While its metabolic derivative, 1-hydroxypyrene, is one of the preferred biomarkers for urinary analysis owing to the strong linear correlation between its urinary levels and the concentration of airborne PAHs [17], parent PAHs are more suitable for adipose tissue biomonitoring. Due to its high lipophilicity (log Kow of approx. 4.88 [18]), pyrene is efficiently sequestered in the triglyceride-rich environment of adipocytes before undergoing metabolic transformation. Hence, studying the morphological and cellular characteristics of pyrene-positive adipose tissue is essential to understand possible early tissue adaptations or toxicity-related alterations.

In this study, we focused on human subcutaneous adipose tissue samples displaying pre-determined pyrene concentrations higher than those reported in the literature (here, from 2.34 to 4.88 ng/g; in the literature, lower than 0.252 ng/g, [9,10,13]); the samples were compared with those from a PAH-negative control. The aim was to provide a comprehensive histopathological characterization of subcutaneous adipose tissue in control and PAH-positive specimens, focusing on overall tissue architecture but also vascular and neural elements and the presence of an eventual inflammatory status. In parallel, adipocytes characteristics, in terms of mean area, were also assessed through a morphometric analysis.

Following preliminary analysis of tissue architecture, H&E staining confirmed the preservation of adipocyte morphology in both control and PAH-positive samples. In fact, the cells exhibited their characteristic appearance, with a round to polygonal shape and nuclei localized at the periphery [19]. These findings suggest that, despite PAH exposure, the fundamental structural integrity of adipocytes remains intact. However, according to morphometric analysis, it was observed that there was a reduction in adipocytes area in PAH-positive samples. This observation can correlate with the role of specific PAHs as environmental obesogens, capable of altering normal adipose tissue development. Compounds such as naphthalene can stimulate preadipocyte proliferation and accelerate early lipogenesis [8,20]. This can yield to smaller, newly formed, yet potentially dysfunctional adipocytes even in the absence of evident structural damage. In parallel, Masson’s Trichrome staining showed well-defined connective tissue septa and comparable collagen distribution in the cohort. Certainly, evaluation of additional samples could strengthen the interpretation of these preliminary findings.

Inflammatory elements were assessed through immunodetection of CD3- and CD68-positive elements. Interestingly, lymphocytic infiltration (CD3) was not evident in the analyzed specimens, suggesting that PAH exposure did not elicit a detectable T-cell-mediated inflammatory response within the hypodermis. Macrophages presence/distribution was verified through CD68 immunostaining. As shown, these cells were mainly localized within the connective tissue septa, associated with blood vessels, where they may contribute to immune surveillance. Additionally, CD68-positive macrophages were recognized among adipocytes, often as isolated elements. This distribution pattern could suggest a basal level of macrophage infiltration within the adipose tissue, potentially involved in maintaining tissue homeostasis or eventually responding to subclinical inflammatory stimuli [21].

S100 and PGP9.5 immunodetection showed the presence of nerve endings at the interface with the hypodermis. The presence of S100- and PGP9.5-positive fibers in association with vascular structures and connective tissue septa may suggest their involvement in neurovascular regulation and mechanosensation. The lower innervation of the hypodermis may be associated with its predominant metabolic function rather than a sensory role [22]. Potential alterations in nerve distribution related to PAH exposure need further investigation with an increased sample size. Additionally, considering the role of adipose tissue innervation in regulating events like lipolysis/thermogenesis/metabolic homeostasis [23,24,25], even minor changes in nerve density or localization could be associated with functional consequences.

Similarly, CD31 staining demonstrated a comparable distribution of endothelial cells/microvessels in both control and PAH-positive samples; the immunopositivity was sparse and associated with connective tissue strands, especially at the dermo-hypodermal interface. Considering the absence of clear vascular remodeling or angiogenesis in PAH-positive samples, any PAH-induced microvascular alterations, if present, are likely to be limited or focal. Moreover, although PAHs have been reported to exert both pro- and anti-angiogenic effects depending on concentration, exposure duration, and metabolic context [26,27,28,29], the preserved vascular architecture observed in the present study does not suggest a major effect on adipose tissue vascularization. Consistently, no significant differences in VCAM-1 expression were detected between groups suggesting the absence of detectable endothelial activation under the conditions examined. Nevertheless, early/minor functional alterations cannot be excluded. Moreover, the association between vessels and fibrous strands needs to be further investigated in order to verify a potential link with PAH-related effects.

Overall, despite study limitations related to the small sample size, the results show a preserved histoarchitecture in subcutaneous adipose tissue despite PAH positivity, with no evident signs of inflammation or angiogenesis. To confirm whether these findings effectively reflect an early tissue adaptation to environmental stress, cohort broadening specifically by increasing the number of PAH-negative specimens, is fundamental. Nevertheless, this study can be intended as a preliminary morphostructural characterization, identifying adipose tissue as a critical site for PAH accumulation and providing a basis for future large-scale epidemiological and histological studies. The study results also raise important questions on the critical threshold/duration of exposure which can induce pathological changes in adipose tissue. Due to the twofold role of the adipose tissue, as both a reservoir of energy and an endocrine organ, even initial histological alterations could eventually lead to systemic metabolic consequences. Future investigations should focus on possible correlations between histological findings with metabolic/transcriptomic profiles; ideally, integrating evidence from both animal models and human biopsies could guide researchers in defining translational impact.

Despite no histopathological alterations being detected in the study cohort, the absence of morphological changes does not exclude the presence of underlying molecular and/or functional disorders. In fact, in accordance with the literature, PAHs can exert subclinical toxicity by activating the Aryl Hydrocarbon Receptor (AhR) pathway with consequent oxidative stress, even in the absence of evident histological damage [30,31]. Specifically, these compounds can trigger early epigenetic modifications, including DNA hypermethylation at key metabolic loci [32,33], which may lead to metabolic reprogramming without immediate structural evidence. As discussed above, the literature broadly highlights the dynamic role of adipose tissue in PAH biodistribution. Despite their sequestration in adipose tissue, which may temporarily reduce PAH bioavailability to peripheral organs, the occurrence of lipolytic states (e.g., weight loss) can trigger their systemic release, with consequent increase in circulating PAH levels and redistribution to metabolically active organs like the liver, heart, and skeletal muscle [34,35]. This mobilization mechanism depends on the activation of lipolytic pathways (adipose triglyceride lipase, hormone-sensitive lipase, and monoacylglycerol lipase) which hydrolyze triglycerides stored in lipid droplets. In fact, while generating glycerol and free fatty acids, this process also promotes the release of co-stored lipophilic compounds from the lipid droplets. Consequently, fluctuations in adipose tissue turnover may modulate internal PAH exposure even without external intake, suggesting that metabolic state has a critical role in determining toxicokinetics and tissue-specific vulnerability even in the absence of histopathological alteration [36]. In turn, increased PAH endogenous exposure can lead to oxidative stress and DNA damage, with possible long-term consequences for cellular and systemic homeostasis. This process may further autocatalyze redox imbalance with sustained reactive oxygen species (ROS) generation [37]. All subjects included in this study were post-bariatric patients. Considering adipose tissue as a long-term reservoir for lipophilic contaminants, including PAHs, the substantial loss of adipose mass following bariatric surgery may promote the release of previously sequestered pollutants into the circulation [38,39]. Although our study was not aimed at monitoring circulating PAH levels or PAH level changes after surgery, their persistence within adipose tissue suggests it as a source of exposure. This aspect may be relevant when interpreting the potential long-term health consequences associated with adipose PAH accumulation. Significant implications have been highlighted for different patient categories, also including children and adolescents, predisposing them to glucose dysregulation, increased blood pressure, and oxidative stress [40,41] and childbearing age obese women, because of potential health risks for the future fetus and breastfeeding infant [42]. Typically, the main PAHs stored within adipocytes are species with lower-molecular-weight (two or three aromatic rings; e.g., naphthalene, phenanthrene); conversely, high-molecular-weight carcinogenic PAHs (four or more aromatic rings; e.g., benzo[a]pyrene, dibenzo[a,h]anthracene), are often below detection thresholds [43,44,45]. In our cohort, a partially overlapping but distinct distribution pattern was observed. In fact, phenanthrene and anthracene were detected inconsistently and frequently below detection limits, whereas pyrene was more consistently measurable across samples. Interestingly, these findings likely suggest cohort-specific variability in PAH retention profiles which may be ascribed to differences in exposure patterns, metabolic processing, or adipose tissue turnover.

Adipose tissue not only stores PAHs but may also have a role in their metabolic activation to carcinogenic metabolites. This process involves multiple pathways [46,47] but a key mechanism is driven by aldo-keto reductases (AKRs) [48,49]. Specifically, human AKR enzymes catalyze the conversion of PAH derivatives into reactive o-quinones, a process that simultaneously generates ROS. O-quinones behave as electrophilic acceptors capable of forming covalent adducts; they are also redox-active, triggering and self-perpetuating redox cycling [50]. Evidence suggests that, by mediating both the sequestration and this metabolic activation of PAHs, adipose tissue directly influences the internal dose, oxidative burden, and metabolic flux of these pollutants [47,48].

Age and body composition have an impact on PAH toxicokinetics. In accordance with physiologically based pharmacokinetic models, neonates and young children have higher internal concentrations of PAHs [51], likely as a consequence of immature xenobiotic metabolism, reduced renal/hepatic clearance, and low adipose mass, with consequent prolonged tissue exposure [52]. In adults, higher total adipose mass may buffer circulating PAH levels, whereas in the elderly, physiological reduction in metabolic clearance increases both internal dose and oxidative stress [53]. This dynamic is particularly significant to our cohort of post-bariatric patients (mean age 48.5 ± 11.8 years); in these subjects, the subcutaneous adipose tissue acts as a substantial reservoir, as demonstrated by the high pyrene concentrations detected (up to 4.88 ng/g) at the time of surgery. Clinical studies in obese populations reinforce the systemic relevance of these pollutants, linking them to metabolic syndrome and endocrine perturbations [8,9]. However, detailed metabolic and endocrine parameters were not available for the present cohort. Although patients underwent rigorous preoperative assessment and subjects with active metabolic syndrome or major uncontrolled metabolic abnormalities were generally excluded according to institutional criteria, the lack of specific biomarkers prevented evaluation of potential associations between adipose pyrene levels and metabolic status, representing a study limitation.

Although some studies suggest that absolute PAH levels can be similar between individuals [54], our data show significant interindividual differences. Beyond these variations in concentration, epidemiological and mechanistic studies suggest that PAH exposure may contribute to systemic metabolic alterations, even without changing the tissue’s appearance [55]. Experimental and human studies on adipose tissue have linked PAH accumulation to specific “not visible” molecular changes, most notably the altered expression of the insulin receptor substrate 2 (IRS2) [32]. This suggests that the high pyrene levels we detected could still impair insulin signaling and metabolic health through the same epigenetic mechanisms that are not captured by standard histological staining. However, markers of oxidative stress, AhR pathway activation, and epigenetic alterations were not investigated in the present study, representing both a limitation and an important direction for future research. Therefore, the possibility of subclinical biological effects cannot be excluded.

Over the last decade, the literature considering the interaction between adipose tissue and PAHs has undergone a significant evolution, moving from the idea of fat as a static storage site up to recognizing it as a key mediator of environmental metabolic toxicity. Initially, research focused on establishing the baseline presence of these pollutants and their sequestration within lipid depots, emphasizing the role of adipose tissue as a primary reservoir [43,48]. Subsequently, research focused on molecular mechanisms associated with PAHs, highlighting their functional effects, including epigenetic changes due to DNA methylation [32], age-related pharmacokinetic differences [53], and their potential obesogenic role [8]. More recently, the field has further broadened, considering systemic health and exploring the eventual influence of stored PAHs on specific pathologies [54,56]. This view recognizes that while the accumulation of PAHs in adipose tissue can contribute to low-grade inflammation, insulin resistance, and endocrine disruption, their release during lipolysis may increase systemic exposure. Therefore, this work, through a critical discussion of experimental evidence with literature data, emphasizes the need to evaluate environmental exposure alongside metabolic status and tissue-specific toxic effects.

Regarding pyrene, research studies on population show that it is a ubiquitous environmental pollutant that can be considered as a critical biomarker for the total burden of low-molecular-weight PAHs in human fat [43]. Detected in over 90% of subjects in biomonitoring studies, pyrene acts as a reliable proxy for combustion-related exposure, rather than heavy industrial sources [9,43]. While its four-ring structure allows it to be sequestered in adipose tissue, which acts as a long-term internal metabolic depot [48,54], it differs from more persistent toxics because it does not show age-dependent accumulation. This suggests that pyrene undergoes active metabolic clearance/excretion, preventing chronic build-up despite continuous environmental exposure [43,53]. Despite its clearance, its strong correlation with other genotoxic PAHs and its accumulation in adipose tissue link it to broader risks, including oxidative DNA damage via AKR enzyme induction [48] and potential metabolic disruptions, such as insulin resistance and pro-adipogenic effects, when released into systemic circulation [8,32]. Within this scenario, a further reflection regards the use of modern anti-obesity pharmacological treatments. As reviewed by Argyrakopoulou et al. [57], molecules such as liraglutide, semaglutide, tirzepatide, naltrexone/bupropion, and bimagrumab effectively reduce total and visceral fat while often preserving or improving lean body mass. Although rapid fat loss can release lipophilic compounds stored in adipose tissue [35], the overall effect is clearly beneficial for patients with metabolic syndrome. The reduction in adipose tissue improves body composition and lowers cardiometabolic risk, overcoming the potential risks of chemical release. Nevertheless, active monitoring of pollutant-related biomarkers during periods of rapid weight loss should be recommended to ensure a safe transition toward metabolic homeostasis.

In conclusion, even though adipose tissue morphostructure appears preserved in the presence of high pyrene levels, without evident signs of inflammation, this should not suggest protection but rather move attention toward metabolic vulnerability during lipid mobilization. Further studies, with a broadened cohort, are required to identify the threshold at which PAHs’ presence translates into permanent tissue damage.

## 4. Materials and Methods

### 4.1. Adipose Tissue Sampling

Adipose tissue samples were obtained from a cohort of 10 patients undergoing surgical procedures, including abdominoplasty, liposuction, prosthesis removal, and mastopexy, after providing written informed consent. Specifically, the samples were preliminarily assessed for PAHs presence, including naphthalene (NA), acenaphthylene (ACL), acenaphthene (AC), fluorene (FL), phenanthrene (PHE), anthracene (AN), fluoranthene (FA), pyrene (Y), benzo[a]anthracene (BaA), chrysene (CHR), benzo[b]fluoranthene (BbF), benzo[k]fluoranthene (BkF), ben-zo[a]pyrene (BaP), benzo[g,h,i]perylene (BgP), indeno [1,2,3-cd]pyrene [IcP], dibenzo[a,h]anthracene [DhP]. The extraction and quantification of PAHs were performed using a QuEChERS-GC-MS methodology recently developed and validated for human adipose tissue [12]. Briefly, the method was optimized using bovine fat as a surrogate matrix due to its biochemical similarity to human tissue (comprising over 90% triglycerides). Validation was conducted on 14 PAHs, yielding Method Detection Limits (MDLs) and Method Quantification Limits (MQLs) ranging from 0.11 to 4.13 ng g^−1^ and 0.38 to 13.76 ng g^−1^, respectively. Matrix effects (ME%) were significantly observed (>20% for most analytes), necessitating the use of matrix-matched calibration curves to ensure quantification accuracy. For pyrene, the specific MDL was 0.31 ng g^−1^ with an MQL of 1.02 ng g^−1^. Trueness values for the most toxicologically relevant compounds were consistent with literature standards for complex lipid matrices. High-molecular-weight PAHs showed robust repeatability (RSD < 20%), confirming the technical reliability of this approach for human biomonitoring. Among the PAHs, PHE, AN and Y were detected, and Y only was also quantified (ng/g) [12] (Table 2). The microscopic and morphometric evaluations were performed on samples with quantifiable Y. The PAH-free specimen #7 was considered as a reference.

Following isolation, the tissues were gently washed in phosphate buffer and then fixed in 10% formalin solution and paraffin-embedded for subsequent histological and immunohistochemical analyses. All participants were enrolled at the Operative Complex Unit of Plastic Surgery, University Hospital of Padua, following approval from the institutional review board (CESC Code: 4502/AO/2018). Informed written consent was also obtained from all participants. Adipose tissue samples were harvested from post-bariatric patients with a BMI ≤ 30, a clinical criterion used to include them in the Plastic Surgery Program. Mean age at the time of surgery was 48.5 ± 11.8 years old.

### 4.2. Histological and Immunohistochemical Characterization

The following analyses were performed only on samples in which contaminants were quantified rather than merely detected.

#### 4.2.1. Assessment of Inflammatory Cells, Blood Vessels, and Nerve Endings

Identification of inflammation-related signs was conducted through light microscopy and immunohistochemical analyses, focusing on lymphocytic infiltration, blood vessels, and nerve endings [58,59,60,61]. To this purpose, 5 μm full-thickness sections from paraffin-embedded samples were dewaxed and rehydrated with a series of ethanol (Arco Scientifica S.r.l., Padua, Italy) solutions (99%, 95%, 70%) and distilled water; hence, sections were stained with H&E. The degree of mononuclear cell infiltration was assessed using the following grading scale: grade 0: no lymphocytic infiltration; grade 1: perivascular mononuclear cell infiltration; grade 2: both perivascular and interstitial mononuclear cell infiltration. For T-cell identification, anti-CD3 immunostaining was performed using a monoclonal mouse anti-human CD3 antibody (code: CD3-565-L-CE, Leica Biosystems, Nussloch, Germany), diluted 1:500 in PBS. Antigen retrieval was carried out with 10 mM sodium citrate buffer (pH 6.0) at 90 °C for 10 min (min). Thereafter, sections were incubated for 30 min at room temperature (RT) in blocking serum [0.04% (*w*/*v*) bovine serum albumin (A2153, Sigma-Aldrich, Milan, Italy) and 0.5% (*w*/*v*) normal goat serum (X0907, Dako^®^, Glostrup, Denmark)] to minimize nonspecific binding. The primary antibody was applied for 1 h (h) at RT. Primary antibody binding was visualized using an anti-rabbit/mouse secondary antibody (EnVision+™ peroxidase, Dako^®^), diluted 1:100 in blocking serum and incubated for 30 min at RT. The reaction was developed with 3,3-diaminobenzidine (DAB) for 3 min, and hematoxylin was used as a counterstain. Negative controls were processed in parallel without the primary antibody.

Macrophages were identified using a monoclonal mouse anti-human CD68 anti-body (code: CD68-L-CE, Leica Biosystems), according to the manufacturer’s protocol.

Nerves and nerve endings were detected using a monoclonal rabbit anti-human S100 antibody (code: S100-167-L-CE, Leica Biosystems) diluted 1:4000 in Dako antibody diluent. The neuronal marker rabbit monoclonal anti-PGP9.5 (code: ab108986, abcam, Cambridge, UK) was used with a 1:300 dilution in Dako antibody diluent to identify nerve fibers.

Vascularization was assessed by analyzing CD31-immunostained sections using a monoclonal mouse anti-human CD31 antibody (code: CD31-607-L-CE, Leica Bio-systems), diluted 1:300. Antigen retrieval was performed in EDTA buffer (high pH) at 90 °C for 10 min. Vascular cell adhesion protein expression was investigated using a rabbit monoclonal VCAM-1 antibody (code: A19131PM, ABclonal, MA, USA) diluted 1:2000 in Dako antibody diluent prior to antigen retrieval with 0.01M Tris-EDTA Buffer (pH 9.0).

Following peroxidase and protein blocking (Dako^®^), sections were incubated with the primary antibody. Detection was achieved using an anti-rabbit secondary antibody (EnVision Flex+ Rabbit, Dako^®^, Glostrup, Denmark), and DAB was used as the chromogen. Hematoxylin was used for counterstaining. Specificity controls included sections processed without the primary antibody.

All peroxidase reactions were repeated at least three times to confirm consistency.

All images were acquired using a Leica D4500B microscope (Leica Microsystems, Wetzlar, Germany) connected to a Leica DC200 high-resolution digital camera (Leica Microsystems).

#### 4.2.2. Collagen Distribution

Masson’s Trichrome staining was performed using a commercial staining kit (Bio-Optica, Milan, Italy) to evaluate the presence and distribution of collagen tissue within adipose tissue samples. Following dewaxing and rehydration, sections were processed according to the manufacturer’s instructions. Briefly, sections were incubated in Weigert’s iron hematoxylin for 10 min, stained with stabilized alcoholic picric acid for 4 min, followed by Ponceau fuchsin for 4 min. After rinsing in deionized water (dH_2_O), slides were incubated in phosphomolybdic acid for 10 min, then stained with light green solution (Goldner’s method) for 5 min. A final rinse in dH_2_O was followed by dehydration in graded ethanol (70%, 95%, 100%) and clearing in xylene. Slides were mounted with Eukitt mounting medium. Images were acquired using a Leica LMD6 microscope (Leica Microsystems) connected to a Leica DFC320 high-resolution digital camera and managed through LasX image acquisition software, version 4.x (Leica Microsystems).

#### 4.2.3. Adipocytes Morphometric Analysis

Adipocytes were manually identified on H&E-stained sections using 10× magnification images. Following image acquisition, photographs were converted to 8-bit binary format, and adipocyte area was manually measured using ImageJ software, version 1.8.0 (NIH, Bethesda, MD, USA). A high-resolution digital camera (DC200, Leica Microsystems, Wetzlar, Germany) was used to capture the images.

For each case, three sequential tissue sections were analyzed, with three non-overlapping fields per section assessed to ensure representative sampling.

### 4.3. Statistical Analysis

Experimental data are expressed as mean ± standard deviation (SD). Statistical analysis was performed using GraphPad Prism version 8.4.2 for Windows (GraphPad Software, San Diego, CA, USA). For multiple group comparisons, one-way ANOVA followed by Tukey’s post hoc test was applied. Statistical significance was set at *p* < 0.05.

## Figures and Tables

**Table 1:** Adipocyte area analysis and comparison.

PAH-Negative (PAH not Detected)		
Specimen	Adipocytes Area (mean values ± SD, µm^2^)	Significance and *p*-Value
**#7**	7633.70 ± 2053.20	** *p* ≤ 0.01 vs. #9; *** *p* ≤ 0.001 vs. #5; **** *p* ≤ 0.0001 vs. #6
**PAH-positive (PAH quantified)**		
**#2**	7942.70 ± 2057.91	** *p* ≤ 0.01 vs. #9; *** *p* ≤ 0.001 vs. #5; **** *p* ≤ 0.0001 vs. #6
**#4**	8088.20 ± 2436.31	*** *p* ≤ 0.001 vs. #9; **** *p* ≤ 0.0001 vs. #5 and #6
**#5**	6437.25 ± 2046.49	*** *p* ≤ 0.001 vs. #8; **** *p* ≤ 0.0001 vs. #6
**#6**	4197.04 ± 1308.25	**** *p* ≤ 0.0001 vs. #8 and #9
**#8**	8108.19 ± 2951.25	*** *p* ≤ 0.001 vs. #9
**#9**	6634.43 ± 2491.62	

**Table 2:** PAHs detected and quantified in human adipose tissue samples. Values are reported in ng/g. Detected analytes are indicated in light red, whereas quantified analytes are highlighted in red. Since sample #7 is completely free from contaminants, it serves as the control and is highlighted in green within the table.

Specimen	Patient BMI	Test	Phenanthrene (PHE)	Anthracene (AN)	Pyrene (Y)
**#1**	25.8	Sample 1	nd	nd	DET
		Sample 2	nd	nd	DET
**#2**	29.5	Sample 1	DET	DET	4.88
		Sample 2	nd	DET	4.81
**#3**	20.3	Sample 1	nd	nd	DET
		Sample 2	nd	nd	DET
**#4**	28.7	Sample 1	nd	nd	3.05
**#5**	28.2	Sample 1	DET	DET	4.73
		Sample 2	DET	nd	3.20
**#6**	27.8	Sample 1	DET	nd	3.61
**#7**	27.3	Sample 1	nd	nd	nd
		Sample 2	nd	nd	nd
**#8**	23.5	Sample 1	nd	nd	2.34
		Sample 2	nd	nd	2.80
**#9**	21.7	Sample 1	nd	nd	DET
		Sample 2	nd	nd	2.74
**#10**	24.8	Sample 1	nd	nd	DET
		Sample 2	DET	DET	2.46

BMI, Body Mass Index; DET, detected; nd, not detected.

## Data Availability

The data presented in this study are available on request from the corresponding author.
